# miRNAs Participate in MS Pathological Processes and Its Therapeutic Response

**DOI:** 10.1155/2016/4578230

**Published:** 2016-03-17

**Authors:** Ting Wu, Guangjie Chen

**Affiliations:** Department of Immunology and Microbiology, Shanghai JiaoTong University School of Medicine, Shanghai Institute of Immunology, Shanghai 200025, China

## Abstract

Multiple sclerosis is the most common autoimmune disease of the central nervous system. It is believed that the increased migration of autoreactive lymphocytes across the blood-brain barrier (BBB) may be responsible for axonal demyelination of neurons. In this review, we discuss microRNAs participating in the pathological processes of MS, including periphery inflammation, blood-brain barrier disruption, and CNS lesions, and in its therapeutic response, in order to find biomarkers of disease severity and to predict the response to therapy of the diseases.

## 1. Introduction

Multiple sclerosis (MS) is the most common autoimmune disease of the central nervous system (CNS) among young adults and it is more common in women than men [1, 2]. It is widely held that there are different four patterns including relapsing-remitting multiple sclerosis (RRMS), primary progressive multiple sclerosis (PPMS), secondary progressive multiple sclerosis (SPMS), and primary-relapsing multiple sclerosis (PRMS) [3].

Although the etiology and pathogenesis of MS remain unknown, several lines of evidence support that the increased migration of autoreactive lymphocytes across the blood-brain barrier (BBB) may be responsible for axonal demyelination of neurons. It has been demonstrated that autoreactive T cells including IFN-*γ*-producing Th1 cells and IL-17-producing Th17 cells may mediate autoimmune in CNS leading to axonal degeneration, demyelination, and ultimately irreversible tissue damage of patients with MS [4, 5]. Treg cells that prevent damage to the host by limiting the immune response to pathogens are impaired in MS [6, 7]. The balance between Th17 and Treg is crucial to the development of MS. Moreover, MS pathology and pathogenesis have been concentrated on extensive demyelination and formation of sclerosis plaques in cerebral cortex and spinal cord, which contribute to irreversible neurological injury. Activated macrophages and microglia are always engaged in active lesions [8]. Both extensive accumulation of macrophages and activation of microglia are common occurrences following neurological injury [9–13]. EAE is a mouse model of the human disease multiple sclerosis, characterized by autoimmune inflammation of the CNS associated with microglia activation and infiltration of encephalitogenic T cells and leukocytes from the periphery [14, 15].

MicroRNAs (miRNAs) are small, usually 19–24 nucleotides in length, noncoding RNAs that regulate gene expression at the posttranscriptional level [16]. Several recent studies have detected the involvement of circulating miRNAs in physiological and pathological processes and identified them as potential biomarkers, therapeutic agents, or drug targets. In MS, a number of miRNA species were found to be differentially expressed in patients with MS compared with controls and to have the potential to be used as diagnostic biomarkers or drug-response.

## 2. miRNAs Involved in Periphery Inflammation

T cell subsets such as Th1 and Th17 accumulate in periphery tissues and mediate adaptive immune response. It is generally believed that Th1 and Th17 cells are key proinflammatory mediators of cellular immunity that are responsible for crucial events during development of EAE. The Th1 lineage cytokine can help Th17 cells invade the brain and spinal, thus triggering EAE [17]. Cytokines produced by DCs may promote CD4^+^ T cell activation and synthesize proinflammatory cytokines.

**Figure 1:**
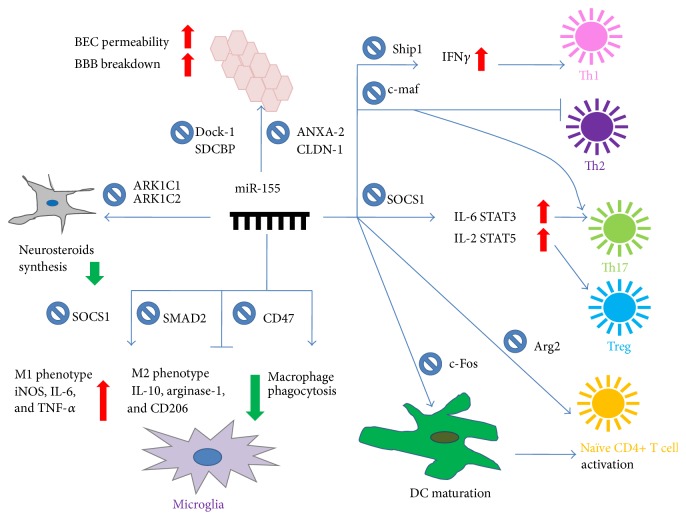
Summary of putative mechanisms through which miR-155 could modulate MS. miR-155 could regulate several inflammatory and anti-inflammatory cytokines through different target genes, participating in MS pathological processes such as periphery inflammation, BBB dysfunction, and CNS lesion.

Several recent studies have examined the involvement of miR-155 in EAE. Besides sustaining inflammatory response by promoting the development of inflammatory Th1 and Th17 cells, miR-155 is critical for the acquisition of phenotypic and functional properties of mature DCs (Figure 1). It is demonstrated that miR-155 modulates IFN*γ* expression through a mechanism involving repression of Ship1, which promotes IFN*γ* level in Th1 cells [18]. An early research reported that miR-155 might block c-Maf, a promoter of Th2 cell development, and enhance Th17 cell differentiation [19]. Recently, miR-155 has been implicated in inhibiting the protein suppressor of cytokine signaling 1 (SOCS1) in activated CD4^+^ T cells, which promotes Treg/Th17 cells differentiation and Th17 function by activating IL-2/STAT5 and IL-6/STAT3 signaling pathways. miR-155−/−mice is associated with a decrease in IL-17 and IFN-*γ*, contributing to a delayed course, decreased EAE clinical severity, and less inflammation in the peripheral lymphoid organs and CNS [20, 21]. Dunand-Sauthier et al. reported that miR155 is required for DC maturation and the ability of DCs to promote antigen-specific CD4^+^ T cell activation by inhibiting c-Fos expression which could dampen TNF-*α*, IL-12p70, IL-6, IFN-*β*, and IL-12p40 secretion by mature BM-DCs [22]. In miR155−/−mices, overexpression of Arg2 in DCs resulted in a selective depletion of arginine which can inhibit T cell responses [23].

miR-146a is highly expressed in Tregs and suppresses interferon (IFN)*γ*-dependent Th1 responses and inflammation by inhibition of its gene expression signal transducer and activator of transcription (STAT-1). The deletion of miR-146a in Tregs may increase the production of IFN-*γ* and develop severe Th1-mediate lesions [24]. Further, miRNA-146a was reported to modulate the signaling proteins involved in the innate immune and inflammatory response, such as complement factor H (CFH) and IRAK-1, and both of them were deficient in MS [25–28].

Overexpression of miR-132 in CD4^+^ T cells from EAE mice downregulated IL-17, IFN-*γ*, and T cell proliferation [29]. miR-26a was demonstrated to downregulate Th17 and to upregulate Treg cell function through targeting IL-6. In EAE, inhibition of miR26a may result in high level of Th17-related cytokines and aggravate clinical signs of EAE. On the contrary, Treg cell specific transcription factor, Foxp3, was detected to be positively correlated with miR26a expression, contributing to a milder form of EAE [30].

A very recent report has shown that miR-21 promoted Th17 differentiation by decreasing SMAD-7, a negative regulator of TGF-*β* signaling. Treatment of wild type mice with anti-miR-21 oligonucleotide diminished EAE clinical severity along with decreased Th17 cells [31].

Guan et al. have reported that upregulation of let-7e leads to promote the development of Th1 and Th17 cells and aggravate EAE. Since, overexpression of let-7e repressed IL-13 and IL-10 production and augmented IFN-*γ* production. Inhibition of let-7e may shift the immune response to a Th2 profile and attenuates the severity of the disease [32].

miR-29ab1 was presented to regulate the Th1 differentiation to affect EAE development by targeting T-bet and IFN-*γ* [33]. Steiner et al. also found that miR-29 repressed IFN-*γ* production by direct targeting of both T-bet and Eomes, two transcription factors known to induce IFN-*γ* production [34]. These results demonstrate that the level of miR-29 can modulate Th1 cell differentiation and reflect the disease severity. A very recent study elucidates that interleukin 6 (IL-6) and RelA (NF-*κ*B subunit) are target genes of miR-291a-3p. Downregulation of miR-291a-3p may indicate oxidative stress at the preonset stage of EAE and upregulation of IL-6 and NF-*κ*B activation to induce proinflammatory pathways by targeting IL-6 and RelA [35].

miR-20b has been reported as a decreased miRNA in blood cells of MS patients. As a negative regulator of EAE, miR-20b suppresses Th17 differentiation* in vitro* and* in vivo* and attenuates EAE by targeting ROR*γ*t and STAT3. This indicates that miR-20b is involved in pathogenesis of EAE mediated by Th17 [36]. Apart from miR-20b, miR-326 [37], miR-301a [38], and miR-23b [39] are found to be involved in Th17-mediated pathogenesis of EAE by targeting Ets-1, a negative regulator of Th17 cell differentiation, IL-6/23–STAT3 pathway, and TAB2, TAB3, and IKK-a, respectively.

**Figure 2:**
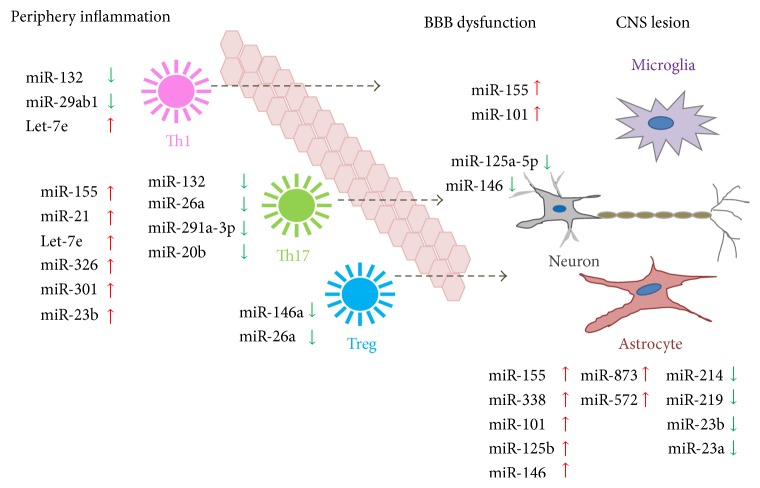
Different miRNA expression in the pathological processes of MS. The changed level of miRNAs is associated with several pathological processes such as periphery inflammation, BBB dysfunction, and CNS lesion. Dysfunction of immune cells including astrocytes and oligodendrocytes in the CNS and Th1, Th17, and Treg cells in the immune system is characterized by different miRNA expressions that are up- (red) and downregulated (green).

In conclusion, these evidences strongly suggest that circulating miRNAs can be utilized as potential clinical biomarkers that reflect the disease activity and severity in MS (Figure 2).

## 3. miRNAs Involved in Blood-Brain Barrier Dysfunction

Disruption and immune activation of the blood-brain barrier (BBB) are central and early features of MS [40–42], with the characteristic of the increased BBB permeability. Previous studies have shown that BBB breakdown is a fundamental event during the course of MS and that the level of the neurovascular dysfunction in EAE may play an important role in the neurological severity of the disease [43].

In recent years, there are increasing evidences to support that miRNAs may play an important role in neuroinflammatory disorders. Several reports have demonstrated that the interendothelial junctional complex (IJC) and integrin focal adhesion (FA) complexes [44–46] are probably associated with altered expression of tight junction (TJ) proteins in response to proinflammatory cytokines, such as TNF-*α* and IFN-*γ*. These cytokines may contribute to increase of brain endothelial cell (BEC) permeability by modulation of its gene expression.

miR-155 is reported as a novel negative regulator of BBB function by modulating BEC cell-to-cell and cell-to-matrix interactions, which contributes to BBB dysfunction in MS. Lopez-Ramirez et al. reported that the putative target genes of miR-155 modulate the alterations in FA and IJC organization that might change the permeability of BEC. There are 4 target genes for miR-155 that have been verified, including 2 components of FAs, DOCK-1 and SDCBP, and 2 components of IJCs, ANXA-2 and CLDN-1, which may modulate BEC permeability and potentially mediate miR-155-induced BBB breakdown during inflammation [47].

During MS, increased permeability and expression of cell-adhesion molecules on the brain endothelium facilitate encephalitogenic T cells and circulating leukocytes infiltrating into the CNS, leading to demyelination and axonal loss [8, 48].

It was recently found that miR-125a-5p may significantly increase BEC barrier function by forming thicker and more continuous junctional complexes of VE-cadherin and zona occludens-1. Overexpression of miR-125a-5p in the brain endothelial cells downregulates TNF-*α*-mediated ICAM-1 expression, a cell-adhesion molecule involved in vascular permeability and leukocyte infiltration, and reduces the transmigration of monocyte through the brain endothelial cell barrier. Therefore, upregulation of miR-125a-5p could reestablish normal function of the brain vasculature in endothelial cell-based neurological diseases, particularly in MS. miR-101 has been shown to downregulate claudin-5 expression by targeting of VE-cadherin, which demonstrates a new mechanism for the regulation of barrier permeability though posttranscriptional regulation of VE-cadherin [49]. Wu et al. investigated that an increasing level of miR-146a expressed on hCMEC/D3 cells diminished cytokine-stimulated adhesion of T cells to endothelial cells, nuclear translocation of NF-*κ*B, and expression of adhesion molecules [50].

In summary, the mechanism of action of miRNA in the inflamed BBB has still not been systematically studied. It is believed that miRNAs such as miR-125a-5p, miR-155, miR-101, and miR-146a can potentially be regarded as novel biomarkers or therapeutic targets for effective treatment of MS (Figure 2).

## 4. miRNA Involved in CNS Lesions

Several recent studies have examined the involvement of circulating miRNAs in microglia activation and demyelination.

miR-873 induced by IL-17 is reported to facilitate the production of inflammatory cytokine and aggravate demyelination in EAE through the A20/NF-*κ*B pathway. Inhibiting miR-873 or A20, respectively, decreased or increased the production of inflammatory cytokines and attenuated or aggravated the CNS damage of EAE mice, both* in vitro* and* in vivo* [51].

A recent study has shown that miR-572 are increased in MS patients [52]. The expression level of miR-572 was varied between patients with different patterns of MS. The serum concentration of miR-572 was lowest in PPMS and it was significantly increased in SPMS. Since a putative target for miR-572 is the neuronal cell-adhesion molecule (NCAM), a protein involved in the maturation of the nervous system [53], the decreased level of miR-572 can promote remyelination in CNS.

It has been generally accepted that the capacity of microglia to phagocytose degenerated myelin can be altered by environmental inflammatory mediators, such as IFN-*γ*, TNF-*α*, IL-4, and IL-10. TNF-*α* was shown to increase the phagocytic activity of microglia. IL-4 and IL-10 exerted a role of upregulating phagocytosis in macrophages/microglia, while accompanied by a reduction of inflammatory response [54].

miR-155 is widely considered as a proinflammatory miRNA. It can target anti-inflammatory proteins in microglia, such as the suppressor of SOCS-1, leading to the upregulation of several inflammatory cytokines, including the inducible nitrogen synthase (iNOS), IL-6, and TNF-*α* related to the M1 phenotype [55]. As a feedback mechanism to control the immune response, it can also upregulate IFN-*β* which increase the expression of SOCS-1 and IL-10, two important anti-inflammatory mediators [56, 57]. In addition, miR-155 can also target M2-associated genes, such as SMAD2, a protein involved in the TGF-*β* pathway [58] and CEBP*β*, a transcription factor critical for the expression of IL-10, arginase-1, and CD206 [59]. Therefore, miR-155 has a positive effect in promoting inflammatory response and upregulating phagocytosis in microglia.

ARK1C1 and ARK1C2 are different isoforms of an enzyme encoding a 3-*α*-hydroxysteroid dehydrogenase activity that is necessary to the synthesis of neurosteroids in the brain. These genes are experimentally validated to be downregulated by miR-155 and miR-338, which can reduce the production of neurosteroids synthesis [60, 61].

Miller and Streit recently demonstrated that decreasing CD47 which is regarded as a protein that inhibits macrophage phagocytosis may promote phagocytosis of myelin in a SIRP-*α*-dependent mechanism [62]. Besides, miR-155-mediated downregulation of CD47 is thought to release macrophages inhibition and thereby promote myelin breakdown [63].

The overexpression of MiR-101 responses to several TLR ligands in macrophages will downregulate MAPK phosphatase 1 (MPK-1), promoting the activation of MAPK and the level of M1-associated proinflammatory cytokines, such as IL-6, TNF-*α*, and IL-1 [64]. Chaudhuri and colleagues reported an increased miR-125b in M1 macrophage activation, with upregulation of MHC class II, CD40, CD80, and CD86. The potential reason is that interferon regulatory factor 4 (IRF4) is targeted by miR-125b and makes macrophages polarize to M1 cytotoxic phenotype [65].

Cui et al. reported that miR-146a-mediated downregulation of IRAK1 is associated with the reinforcement of IRAK2-induced activation of NF-*κβ* and a sustained inflammatory response in human astroglial cells [26]. Interestingly, significant amounts of miRNA-146a that have been found in glial cells are responsible for axonal myelination [66–68]. In inactive MS lesions, miR-219 are the most downregulated miRNAs [63]. The enzyme ELOVL7 regulated by miR-219 is essential for myelin maintenance and axonal integrity in the adult mouse CNS [69].

In recent years, there are increasing evidences to support that some miRNAs are engaged in building beneficial environment for remyelination and axon regeneration. For instance, two studies described that miR-214 is upregulated in oligodendrocytes during differentiation and miR-23a overexpression promotes oligodendrocyte differentiation by downregulating lamin B. The increased level of miR-214 and miR-23a in MS lesions may reflect ongoing remyelination [70, 71]. The microglia in CNS are the main macrophages participating in phagocytosis in the early stage of demyelination, while in the late stage, a number of infiltrated blood-borne macrophages contribute to axon debris clearance.

miR-124 is reported as a key regulator of microglia quiescence in the CNS and as a new modulator of monocyte and macrophage activation in the periphery during EAE. miR-124 has been reported to contribute to the M2 phenotype of macrophages and microglia, since its overexpression led to the downregulation of M1-associated markers, such as IL-6, TNF-*α*, and iNOS, and an increase of proteins associated with the M2 phenotype, such as TGF-*β*, arginase-1, and FIZZ1 [72], which are crucial for the suppression of EAE. In EAE, Zhu et al. have experimentally validated TAB2, TAB3, and IKK-*α* as miR-23b targets. These genes are upregulated during EAE and modulate IL-17, TNF-*α*, and IL-1*β* induced activation of NF-*κβ*. These findings indicate that miR-23b overexpression can delay the onset of EAE and alleviates disease severity [39] (Figure 2).

## 5. miRNA Involved in MS Therapeutic Response

Natalizumab is a recombinant humanized monoclonal antibody which binds to *α*4*β*1 and *α*4*β*7 integrins and suppresses their interaction with vascular cell-adhesion molecules-1 (VCAM-1) so as to impair leucocyte adhesion and transmigrate across the BBB into the central nervous system, with a reduction of proinflammatory cytokines [73].

Ingwersen et al. reported that five miRNAs (miR-18a, miR-20b, miR-29a, miR-103, and miR-326) were regulated by natalizumab, which were the opposite of the miRNA results in MS patients prior to natalizumab therapy compared to healthy controls. Four of them (miR-18a, miR-20b, miR-29a, and miR-103) were upregulated, while miR-326b is downregulated after natalizumab treatment [74]. As mentioned above, the targets of miR-20b and miR-326, including ROR*γ*t, stat3, vegfa, and Ets-1 [37, 74–76], play an important role in regulating Th17 immune responses and BBB breakdown [77, 78]. Further, miR-29a was shown to modulate the differentiation of proinflammatory Th1 immune responses [33].

Petrocca et al. demonstrated that natalizumab may downregulate miR-17 expression and upregulate the level of miR-106b. Both miR-106b and miR-17 have been identified as important modulators of TGF-*β* signaling [79] and have been found to regulate CD4^+^ T cell immune responses by targeting TGFBR2 [80]. The TGF-*β* pathway is necessary for maintaining peripheral Foxp3-expressing regulatory T cells [81]. Inhibition of TGFBR2 resulted in severe inflammatory responses associated with T cell activation and differentiation in mice [82].

In line with decreased miR-17 in natalizumab treated patients, we found an increase in PTEN mRNA, which plays an important role in the regulation of T cell homeostasis and self-tolerance [83, 84]. In addition, downregulation of miR-17 was associated with upregulation of a proapoptotic member of the Bcl-2 family, BIM, the cyclin-dependent kinase inhibitor 1, p21, and a transcription factor controlling the G1-S transition, E2F1 [85].

These targets reverted by natalizumab therapy may alter peripheral immune cells during EAE and negatively associate with disease severity. So the influences of natalizumab on the expression of miRNA can help us to understand long-term effects of natalizumab and functions in the process of MS pathogenesis.

Interferon-beta (IFN-*β*) therapy is widely used in patients with MS. Besides its effects on immunomodulatory system and metalloproteinase 9 [29], it has been shown to have an effect on neuroprotection against the toxicity induced by activated microglia and inhibiting the production of glutamate and superoxide by activated microglia [86, 87]. It has been reported that the expression level of miR-26a-5p was significantly changed in response to INF-*β* treatment in MS patients during different stages. It was much higher in IFN-*β* treated RRMS patients at 3 months' treatment and kept stable at 6 months' treatments in all patients. In contrast, postsynaptic density protein 95 (DLG4), a key player in neuronal signaling, decreased after 3 months' treatment, showing an inverse relation to miR-26a-5p expression [88].

Moreover, it has been reported that 20 miRNAs may play an important role in the mechanisms of therapy of IFN-*β*. Interestingly, seven of them, including miR-16-5p, miR-342-5p, miR-346, miR-518b, miR-760, let-7a-5p, and let-7b-5p, were increased, whereas 13 miRNAs, including miR-27a-5p, miR-29a-3p, miR-29b-1-5p, miR-29c-3p, miR-95, miR-149-5p, miR-181c-3p, miR-193a-3p, miR-193-5p, miR-423-5p, miR-532-5p, miR-708-5p, and miR-874, were decreased in PBMCs from MS patients in response to IFN-*β* treatment. Particularly, some of the 20 miRNAs, such as members of the mir-29 family, are associated with apoptosis and are involved in IFN signaling feedback loops and others like hsa-miR-532-5p and hsa-miR-16-5p are demonstrated to have relations with IFN-responsive genes [89]. This information can be served as a novel biomarker to predict the effects of IFN-beta therapy and be applied to develop a novel therapy for MS patients.

Glatiramer acetate (GA), an immunomodulation drug, was approved as a first-line therapy in MS [90]. GA treatment may downregulate miR-146a and miR-142-3p level in the PBMCs of MS patients. The mechanisms by which GA influences the expression of miR-142-3p may attribute to an expansion of T regulatory cells and changes in the composition of the T cell compartment [76]. Downregulation of miR-146a in the glatiramer acetate treatment group may improve disease status of MS patients by inducing a Th1 to Th2 shift and inhibiting monocyte reactivity [91–93].

The GSK3*β* inhibitors were shown as beneficial drugs in preclinical trials in stroke and Huntington's and amyotrophic lateral sclerosis, with functions of increasing BBB tightness under physiologic conditions, decreasing inflammatory factors secreted in brain microvascular endothelial cells and protecting against BBB disruption by reducing monocyte adhesion to/migration across the BBB [94, 95]. Rom et al. discovered that the GSK3*β* inhibitor may upregulate miR-98 and let-7g^*∗*^ so as to attenuate leukocyte adhesion/migration into the BBB and diminished BBB permeability in both* in vitro* and* in vivo* models. Overexpression of miR-98 and let-7g^*∗*^ in brain endothelium may also contribute to inhibiting expression of proinflammatory mediators, such as CCL2 and CCL5 [96].  Table 1 summarized miRNA biomarkers involved in the therapeutic response in multiple sclerosis.

## 6. Conclusion

MS is a chronic and systemic autoimmune disease with different disease stages. Different patterns of patients with MS undergo several disease processes including periphery inflammation and blood-brain barrier damage and CNS lesions. Without timely and adequate treatment, these patients will suffer chronic demyelination and axonal loss, for unclear reasons, leading to irreversible disability. The discovery of MS biomarkers may extremely improve the diagnosis and management of MS. The current miRNA profiles offer an opportunity to indicate disease progression and the therapeutic effect. Some of the biomarkers are related to an altered biological process and a treatment targeting this process. Understanding the complexity of miRNA network may open up a new vista to find individual biomarkers in clinical diagnosis and monitor the efficacy of therapy.

## Figures and Tables

**Table 1:** MiRNA biomarkers of therapeutic response in multiple sclerosis (MS).

Drug treatment	Increased miRNA	Decreased miRNA
NatalizumAb	miR-18a, 20b, 29a, 103	miR-326, 17

INF-*β*	miR-26-5p, 16-5p, 342-5p, 346, 518b, 760, let-7a-5p, 7b-5p	miR-27a-5p, 29a-3p, 29b-1-5p, 29c-3p, -95, 149-5p, 181c-3p, 193a-3p, -193-5p, 423-5p, 532-5p, 708-5p, 874

GA		miR-146a, miR-142-3p

GSK3*β* inhibitors		miR-98 and let-7g^*∗*^
